# Systematic and morphometric data of late Miocene rodent assemblage from Triblavina (Danube Basin, Slovakia)

**DOI:** 10.1016/j.dib.2019.104961

**Published:** 2019-12-07

**Authors:** Peter Joniak, Michal Šujan

**Affiliations:** Comenius University in Bratislava, Department of Geology and Paleontology, Ilkovičova 6, Mlynská dolina G, SK–842 15 Bratislava, Slovakia

**Keywords:** Small mammals, Rodentia, Turolian, Central Europe

## Abstract

The lower Turolian (MN11) assemblage of rodents collected from new temporal outcrop near Triblavina (Danube Basin, Western Slovakia) is described. Fossils were collected from at least 2.5 tons of sediment that was wet sieved on a set of stable sieves. The mesh size of the lower sieve was 0.5 mm and the residue was not treated with acids for the sake of preserving mollusk shells. Fossil remains were picked manually from residue under the stereo-microscope. More than 170 teeth of small mammals were recovered. The rodent assemblage consists of 87 identifiable teeth belonging to ten taxa: *Apodemus lugdunensis, Kowalskia* sp., *Epimeriones austriacus, Eozapus intermedius, Spermophilinus* sp., *Myomimus dehmi, Vasseuromys pannonicus, Keramidomys ermannorum, Keramidomys* sp. and *Eomyops* sp. Fauna of rodents from Triblavina is described in detail here and these data are associated with recent article [1].

**Table:** Specifications Table

Subject	Palaeontology
Specific subject area	Taxonomy and systematic of rodent assemblage
Type of data	Text Table Image
How data were acquired	The specimens were collected from the temporal outcrop near Triblavina in June 2017. At least 2.5 tons of sediment from the fossiliferous layer was dried, soaked and washed on a set of stable sieves. The mesh size of the lower sieve was 0.5 mm and the residue was not treated with acids for sake of preserving mollusc shells. Fossil remains were then picked manually under the stereo-microscope. Teeth measurements in mm were taken by calibrated micrometer eyepiece on a Leica MZ75 stereomicroscope. Photos were taken on QUANTA™ SEM microscope.
Data format	Raw (Microsoft Excel file) Analyzed (within the text)
Parameters for data collection	Standard conditions during field campaign and laboratory process.
Description of data collection	Each specimen was measured with precision 0.01 mm using calibrated micrometer eyepiece on a Leica MZ75 stereomicroscope. Morphological descriptions are based on all available specimens. Representative specimens were used for figures.
Data source location	City/Town/Region: Triblavina, Bratislava district Country: Slovakia Latitude and longitude (and GPS coordinates) for collected samples/data: 48°12′50.61″N, 17°15′38.47″E
Data accessibility	With the article Material is housed in repository of: Comenius University in Bratislava Department of Geology and Paleontology Ilkovičova 6, Bratislava, Slovakia Data identification number: PRIFUK/TB170000–TB170048; TB170060–TB170113; TB170119; TB170120. Raw data: https://doi.org/10.17632/d7jfd652rb.1
Related research article	Peter Joniak, Michal Šujan, Klement Fordinál, Régis Braucher, Samuel Rybár, Marianna Kováčová, Michal Kováč & AsterTeam The age and paleoenvironment of a floodplain alongside late Miocene Lake Pannon: Rodent and mollusk biostratigraphy coupled with authigenic^10^Be/^9^Be dating in the northern Danube Basin (Slovakia). Palaeogeography, Palaeoclimatology, Palaeoecology. https://doi.org/10.1016/j.palaeo.2019.109482

**Table:** 

**Value of the Data** • The dataset is first description of the new small mammal fauna recovered from Triblavina outcrop. The composition of the rodent assemblage can be used for biostratigraphical, palaeoecological and palaeobiogeographical interpretations. • The data are important especially for many palaeontologist dealing with evolution, morphometry and taxonomy of small mammals as well as for all specialist dealing with biostratigraphy of Miocene terrestrial deposits. • Data provided here can be used for comparison with material from other localities for better understanding of evolution of Miocene rodents, for geochronology and for palaeoenvironmental interpretations.

## 1 Data

Raw data are available here: https://doi.org/10.17632/d7jfd652rb.1.  

Order Rodentia Bowdich, 1821

Family Muridae Illiger, 1811

Subfamily Murinae Illiger, 1811

Genus *Apodemus* Kaup, 1829

*Apodemus lugdunensis* (Schaub, 1938)

(Fig. 1A-F)

**Fig. 1:**
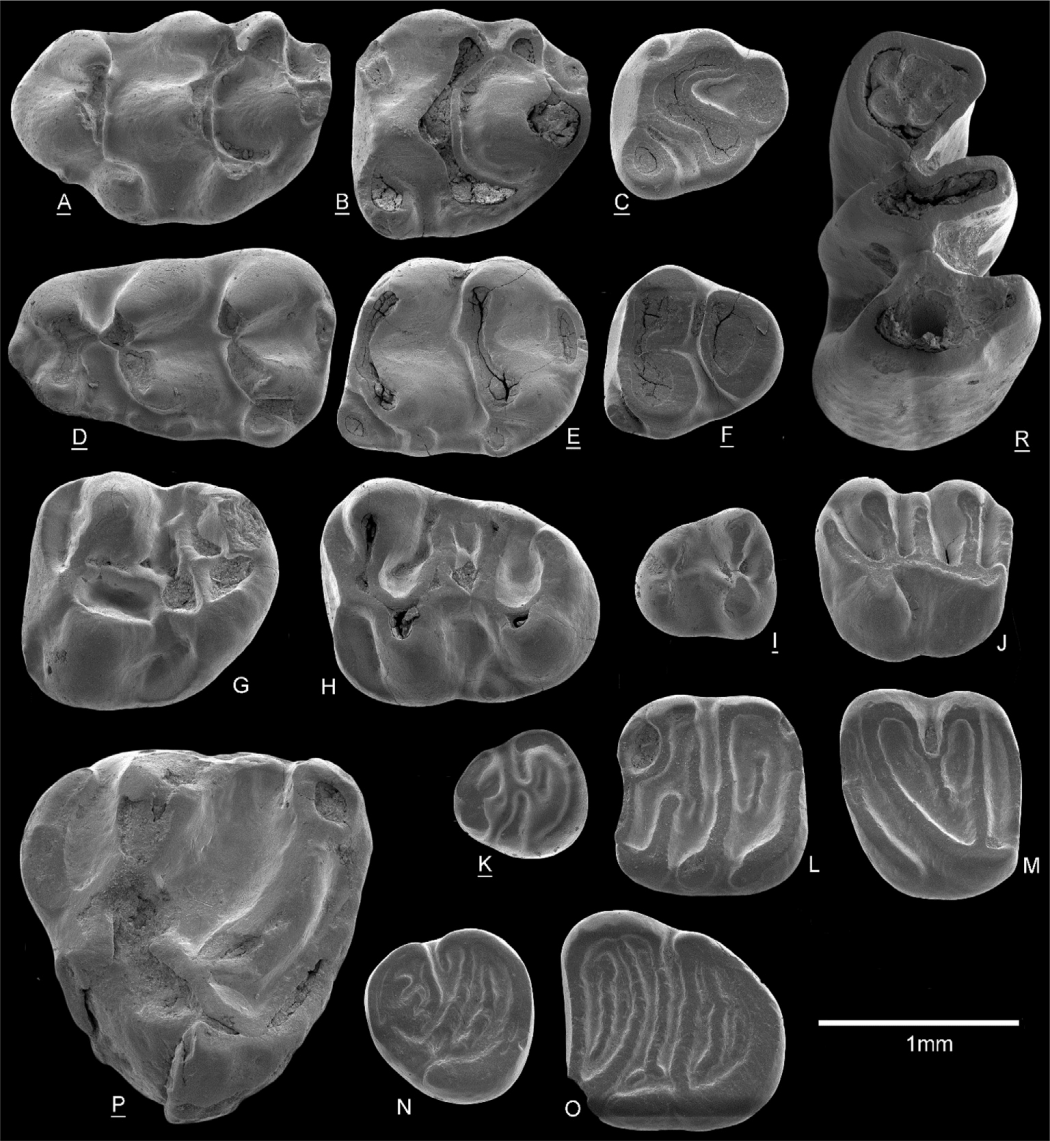
Teeth of rodents from Triblavina. **A-F**. *Apodemus lugdunensi****s*, A**. M1 dex. (TB170001), **B**. M2 dex. (TB170018), **C**. M3 dex. (TB170024), **D**. m1 dex. (TB170033), **E**. m2 dex. (TB170039), **F**. m3 dex. (TB170047). **G-H.***Kowalskia* sp., G. M3 sin. (TB170061), **H**. m3 sin. (TB170063). **I**. *Eozapus intermedius*, M1 sin. (TB170070). **J**. *Eomyops* sp., p4 dex. (TB170118). **K-M**. *Myomimus dehmi*, **K**. p4 dex. (TB170077), **L**. m1 sin. (TB170078), **M**. M1 sin. (TB170079). **N–O**. *Vasseuromys pannonicus,***N**. p4 sin. (TB170704), **O**. m3 sin. (TB170076). **P**. *Spermophilinus* sp., P4 dex. (TB170120). **R**. *Epimeriones austriacus,* m1 dex. (TB170065). Reversed specimens are underlined.

Material – 44 isolated molars. 13 M1 (TB170000-0012), 5 M2 (TB170014-0018), 7 M3 (TB170019-0025), 6 m1 (TB170030-0033, 0036, 0037), 5 m2 (TB170034,0035, 0038-0040), 8 m3 (TB170041-0048). Measurements of isolated cheek teeth are in Table 1.

**Table 1:** Teeth measurements of *Apodemus lugdunensis* from Triblavina [in mm].

	L			N	W		
	min	mean	max		min	mean	max

M1	1.61	1.69	1.84	8/9	1.04	1.10	1.16
M2	1.13	1.20	1.28	4/5	0.98	1.08	1.17
M3	0.81	0.84	0.89	6/4	0.81	0.84	0.91
m1	1.65	1.67	1.68	3/3	0.92	0.99	1.03
m2	1.17	1.22	1.28	5/5	1.03	1.04	1.06
m3	0.91	0.93	0.97	7/7	0.78	0.84	0.89

Description: M1 – the t1 is placed posteriorly to t2 and t3; t1bis and t2bis are absent. The t7 is absent as distinctive cusp but the crest between t4 and t8 is present in all but one specimen. In this specimen, this crest is present but is interrupted. The t6 and t9 are connected in all specimens. The t12 is developed as a small cusp.

M2 – the t1 is large relative to t3. The t1 is a rounded cusp in three specimens, while it is elongated and split in two others. The latter two specimens (TB170014 and TB170015) probably came from the same animal. Both t1 and t3 are connected to the anterior base of the t5. The t4 is connected to t8 by a low crest. The t7 is absent and t12 is well developed.

M3 – The t1 is connected to t5 in five specimens, while it is isolated in two. The t3 is small and in three specimens developed only as a low ridge. The cusps t4-t5-t6-t8 are connected and t9 is reduced or fused with t8. The connection between t4 and t8 is weak or absent. The t12 is not developed.

m1 – The antero-central cusp is present in all specimens. The anteroconid and metaconid are connected in three specimens; in one specimen, these cusps are separated but weak unconnected spurs on the anteroconid and metaconid are present. The longitudinal crest is absent and the terminal heel is elongated. The labial accessory cusps are well developed. The posterior accessory cusp is larger than anterior ones and connects to the hypoconid.

m2 – The antero-labial cusp is rounded in four and crest-shaped in one specimen. The longitudinal crest is absent on all, but in one specimen it is short and low. The terminal heel is oval. The labial accessory cups are usually two, the posterior being larger and connected to the hypoconid in three specimens while separated in two others.

m3 – The antero-labial cusp is small, ridge-shaped and connected to the protoconid. The protoconid and the metaconid are connected, but separated from the hypoconid and entoconid. Latter cusps are distinctive only in young and unworn specimens; in older, they are fused into one elongated or rounded cusp.

Remarks: Murids represent the most common element in the rodent assemblage from Triblavina. The teeth show a morphology that can be clearly attributed to early species of genus *Apodemus*, e.g. absence of t7 and instead development of ridge connecting t4 and t8 in the upper molars; connection of t6 and t9; development of t12. In the lower molars, it is mainly the absence of longitudinal crest, presence of the antero-central cusp and development of labial accessory cusps. These morphological features allow assigning these teeth to *Apodemus lugdunensis*. Moreover, the sizes of the available teeth clearly fit with the size range of this species.

Further comparison with known populations from surrounding areas, especially from Austria, shows that our *Apodemus* is morphologically closer to the populations from Eichkogel and Kohfidisch (both MN11) than to those from Schernham (MN10). Younger populations show a higher percentage of t6-t9 connection of M1 and M2, higher frequencies of presence of the anterocentral cusp of m1 and well-developed labial accessory cusps of m1 and m2 [2]. Moreover, the M3 show a pattern more similar to younger populations from Eichkogel and Kohfidisch in the reduction of t9 and in the development of the connections between t8 and t4.  

Subfamily Cricetinae Fischer, 1817   

Genus *Kowalskia* Fahlbusch, 1969

*Kowalskia* sp.

(Fig. 1G-H)  

Material: 5 isolated molars. 3 M3 (TB170060 1.26 × 1.29, TB170061 1.28 × 1.21, TB170062 1.20 × 1.13), 2 m3 (TB170063 1.42 × 1.14, TB170064 – x –).

Description: M3 – All specimens show complex but homogenous dental pattern. The lingual anteroloph is short and weak. The anterior protolophule connects to the short anterolophule. The posterior protolophule is longitudinal and forms the axioloph. The neo-entoloph is complete and the mesoloph is long. The metacone is small but prominent and the forward directed anterior metalophule connects to the mesoloph. The posterior metalophule connects to the strong posteroloph.

m3 – the anterolophids are developed equally in length but labial anterolophid is low and connects to the base of the protoconid. The mesolophid is long and parallel with the hypolophulid. The entoconid is small but distinctive and separated from oblique and thick posterolophid. The ectostylid is developed close to the base of the hypoconid in the sinusid.

Remarks: There is no consensus in the synonymy of *Kowalskia* and *Neocricetodon* suggested by Freudenthal et al. [3]. Daxner Höck & Höck [2], Sinitsa & Delinschi [4] and references therein contain the most recent references, and here we follow the Daxner-Höck & Höck [2] use of *Kowalskia* in the European record.

The third hamster molars are usually reduced and show significant morphological variation. Our collection involves only these elements thus identification is rather tricky. Our molars are relatively small and comparable in size with those of *K. skofleki* from Eichkogel and they are smaller than *K. fahlbuschi* from Kohfidisch. The comparison with *Kowalskia* sp. A, *K.* sp. B and *K.* sp. C from Austrian localities [2] is difficult because metrical data are not available. On the base of description and figured specimens *Kowalskia* sp. B and smaller specimens of *Kowalskia* sp. C seem to be comparable with material from Triblavina. The posterior part of our upper molars is less reduced than in *K. skofleki* and in this respect resembles more *K. fahlbuschi* from Kohfidisch and *Kowalskia* sp. C from Schernham. Moreover, available lower molar from Triblavina shows separated posterolophid from relatively small entoconid – a feature that seems to be more characteristic for *K. fahlbuschi* and *Kowalskia* sp. C. The limited hamster material from Triblavina clearly belongs to *Kowalskia* but further identification is impossible so far.  

Family Dipodidae Fischer 1817   

Subfamily Zapodinae Coues, 1875

Genus *Eozapus* Preble, 1899

*Eozapus intermedius* (Bachmayer & Wilson, 1970)

(Fig. 1J)  

Material: 3 isolated molars. 1 M1 (TB170070 1.01 × 0.96), 1 M2 (TB170071 1.05 × 0.89), 1 m2 (TB170072 1.07 × 0.80).

Description: M1 – the anterocone is not developed, but the long anterolophule is reaching antero-labial part of the tooth. The protocone is a rounded cusp, while the hypocone is wide and transversely compressed. The mesoloph is long and strong. The paracone and metacone are higher than the protocone and the hypocone. The protoloph connects to the posterior edge of the protocone and the metalophule connects to the middle of the hypocone. The entoloph is complete and the posteroloph is long and straight.

M2 – the general morphology is similar to that of the upper first molar, but the protocone and the hypocone are both transversely compressed and are equally wide. The endoloph is complete. The protoloph connects to the protocone more anteriorly and the posteroloph is more oblique than in M1.

m1 – the lingual anterolophid is longer than the labial one. The metalophulid is curved anteriorly. It connects to the anterolophid but not to the protoconid. The protosinusid is open and continuous with lingual synclinid II. The connection between the protoconid and mesoconid is constricted. The mesoconid is placed centrally and the mesolophid is long. The hypolophulid has weak anterior connections to the mesoconid and to the hypoconid. The posterolophid is strong, oblique and separated from the entoconid.

Remarks: The teeth of *Eozapus intermedius* from Triblavina match in size and fit also perfectly in morphology with the type material of *E. intermedius* described by Bachmayer and Wilson [5] from Kohfidisch (MN11).  

Subfamily incertae sedis  

Genus *Epimeriones* Daxner-Höck, 1972

*Epimeriones austriacus* Daxner-Höck, 1972

(Fig. 1R)  

Material: 2 isolated teeth. 1 m1 (TB170065 1.93 × 1.18), 1 m2 (TB170066 1.35 × 1.20), 3 fragments.

Description: m1 – unworn tooth with three anticlines and two synclines on each side. The tooth is wider posteriorly. The labial side of the anterior lobe is more oblique than the lingual side. The occlusal surface of the anterior lobe still has remains of enamel. The middle lobe is narrower than the anterior and posterior lobes. The posterior lobe has a shallow indentation on its postero-lingual side. The synclinids are deep.

m2 – the tooth is worn and shows a bilobate pattern with synclinids on each side. The anterior lobe is wider than the posterior lobe and has a shallow indentation on the antero-lingual side. The posterior lobe has an oblique posterior margin.

Remarks: The size and morphology of our specimens is identical to the type population of *Epimeriones austriacus* from Eichkogel (MN11). In spite of having only two specimens available from Triblavina, our material can be attributed to this species with certainty because of the characteristic morphology.  

Family Gliridae Muirhead, 1819  

Genus *Myomimus* Ognev, 1924

*Myomimus dehmi* (de Bruijn, 1966)

(Fig. 1K-M)  

Material: three isolated molars. 1 M2 (TB170079 0.68 × 0.65), 1 p4 (TB170077 0.99 × 1.03) and 1 m1 (TB170078 0.98 × 1.07).

Description: M2 - the shape of the concave occlusal surface is sub-rectangular, but the anterior part is slightly narrower than the posterior part. The anteroloph is separated from the protocone and from the metacone. The protoloph and the metaloph are complete and connected to the main cusps. The anterior centroloph and posterior centroloph are fused in the middle of the occlusal surface; thus, they form with the protoloph and metaloph a heart-shaped structure. The posteroloph is long and connects the protocone to the base of the metacone. The M2 has three roots.

p4 – The outline of the occlusal surface is oval. The anterolophid is divided into a labial and lingual branch by a small valley on its anterior wall. A short longitudinal ridge runs posteriorly from the anterolophid. The metalophid is absent and the mesolophid is interrupted. A short posterior extra ridge is present. The p4 has one root.

m1 – The outline of the m1 is sub-rectangular. The anterior part is slightly narrower than the posterior part. The anterolophid connects labially to the metalophid and lingually to the metaconid. The metalophid is interrupted in the middle; its lingual portion is curved strongly anteriorly and connects to the anterolophid. The centrolophid is lingually constricted before the metaconid and ends free labially. The endolophid is interrupted between the centrolophid and the mesolophid. The mesolophid and the posterolophid incorporate the entoconid, forming a U shape posterior valley. The posterior extra ridge is low but well developed. The m1 has three roots.

Remarks: The most striking character of *Myomimus* are the three-rooted lower molars which make these teeth distinctive from those of *Peridyromys* and *Miodyromys*, which have an overall similar dental pattern, but two roots only. For determination on the species level is important presence of a fused centroloph, a feature that appears more frequently in populations of *M. dehmi* than in populations of *M. maritsensis*, but never in *M. quafzenis* [6]. The dental pattern of lower premolar is comparable with premolars of *M. dehmi* from Nombrevilla (MN9, Spain) and first lower molar from Triblavina has, similarly as Austrian specimens from Eichkogel (MN11) and Kohfidisch (MN11) [2], a slightly more complicated pattern than Spanish population.  

Genus *Vasseuromys* Baudelot & de Bonis, 1967  

*Vasseuromys pannonicus* (Kretzoi, 1980).

(Fig. 1N-O)  

Material: three isolated molars. 1 p4 (TB170074, 0.87 × 0.89), 1 m1 (TB170075, 1,21× –) and 1 m3 (TB170076, 1.14 × 1.12).

Description: p4 – the outline of the tooth surface is rounded and narrower in its anterior part. The dental pattern consists of the anterolophid that is lingually connected to the curved metalophid and is labially connected to the labial end of the mesolophid. There are two irregular short ridges in the valley between the anterolophid and the metalophid. The posterior part of the tooth consists of the strong posterolophid, mesolophid, posterior valley extra ridge and a short extra ridge in the central valley. The endolophid is interrupted.

m1 – the tooth is worn and has a damaged labial margin. The endolophid is interrupted. The anterolophid and the metalophid are labially connected. The labial parts of the metalophid, centrolophid, mesolophid, posterior valley extra ridge and posterolophid are oriented parallel to one another. The central part of the tooth is worn, but remains of several irregularly oriented ridges are recognizable. The roots are not preserved.

m3 – the tooth has small damage on its antero–labial part. The tooth is narrower in its posterior part. The tooth pattern consists of eight ridges and two very low extra ridges in narrow valleys. The anterolophid and the metalophid are labially connected. The labial ends of the mesolophid and posterolophids are connected, thus forming an incomplete ectolophid. Two extra ridges are developed in the posterior valley. The centrolophid is long and labially forked. The endolophid is interrupted.

Remarks: *Vasseuromys pannonicus* is characteristic by very complicated pattern with many irregularly developed ridges and extra ridges. This species is known in central Europe from the Austrian localities of Eichkogel (MN11) and Kohfidisch (MN11) and from the Hungarian locality Széchenyi hills in Budapest where it was described by Kretzoi [7] as *Szechenyia pannonica*.  

Family Sciuridae Fisher, 1817  

Subfamily Sciurinae Fisher, 1817

Genus *Spermophilinus* de Bruijn & Mein, 1968

*Spermophilinus* sp.

(Fig. 1P)  

Material: one P4 (TB170120 1.78 × 1.98).

Description and remarks: The enamel of the anterior wall of the protocone is slightly damaged. The protoloph and metaloph are high ridges, while anteroloph and posteroloph are worn down to a much lower level. The metacone and the paracone are distinct. The metaconule is developed as a small protuberance. The mesostyle is weak. The size and the main morphological features allow attribution to the genus *Spermophilinus*, but further identification of this partly damaged upper premolar is not possible.  

Family Eomyidae Depéret & Douxami, 1902  

Genus *Eomyops* Engesser, 1979

*Eomyops* sp.

(Fig. 1I)  

Material: one isolated p4 (TB170118, 0.72 × 0.68).

Description and remarks: The anterior part of the tooth is narrower than the posterior part. The protoconid is slightly larger than the metaconid, but both cusps are weaker than the hypoconid and entoconid. The metalophid is short and connects to the protoconid. The hypolophid connects to the hypoconid. The mesolophid is only outlined and the posterolophid is strong but short. The sinus is widely open.

Although only one lower premolar was found, the morphology of the tooth is well comparable to lower premolars of *Eomyops*. The size of our specimen is smaller than *E. catalaunicus*, but is comparable with *E. oppligeri* from Anwil [8]. Because the morphology of lower premolars can be similar in various species of genus *Eomyops*, we prefer to leave the species determination open.  

Genus *Keramidomys* Hartenberger, 1966  

*Keramidomys ermannorum* Daxner-Höck & Höck, 2009

(Fig. 2A-H)

**Fig. 2:**
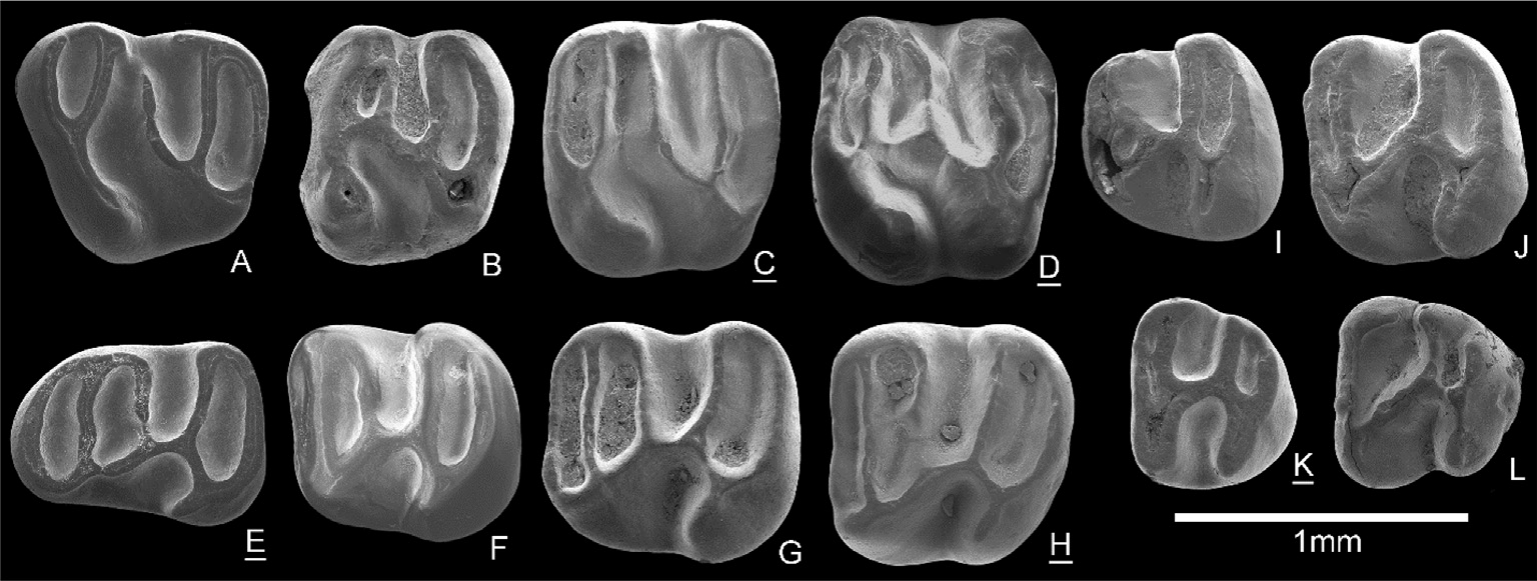
Teeth of rodents from Triblavina. **A-H**. *Keramidomys ermannorum,***A**. D4 sin. (TB170080), **B**. P4 sin. (TB170084), **C**. M1 dex. (TB170087), **D**. M1 dex. (TB170088), **E**. d4 dex. (TB170100), **F**. m1 sin. (TB170104), **G**. m1 sin. (TB170105), **H**. m2 dex. (TB170106). **I-L**. *Keramidomys* sp., **I**. p4 sin. (TB170102), **J**. m1/2 sin. (TB170103), **K**. m3 dex. (TB170110), **L**. m3 sin. (TB170112). Reversed specimens are underlined.

Material: 2 D4 (TB170080, 0.86 × 0.84; TB170081, –x–), 3 P4 (TB170082, 0.83 × –; TB170083, – x –; TB170084, 0.73 × 0.83), 1M1/2 – (TB170085, 0.83 × 0.98; only outline is preserved), 4 M1 (TB170086, 0.81 × 0.91; TB170087, 0.84 × 0.90; TB170088, 0.86 × 0.94; TB170089, 0.87 × 0.93), 2 M2 (TB170090, 0.77? x 0.92; TB170091, 0.65 × 0,81), 2 d4 (TB170100, 0.89 × 0.64; TB170101, – x 0.63), 2 m1 (TB170104, 0.80 × 0.77; TB170105, 0.90 × 0.85), 3 m2 (TB170106, 0.85 × 0.85; TB170107, – x 0.82; TB170108 – x –).

Description: D4 –one complete specimen has a trapezoidal occlusal surface. The anteroloph and the protoloph are both about the same length and they connect to the crest-shaped protocone. The mesoloph is long; in one specimen, it connects to the paracone, while in other is interrupted before it reaches paracone. The longitudinal crest is interrupted. The 1st and 4th synclines are closed.

P4 – The protoloph is absent in all specimens. The mesoloph is short in one while long and connected to the paracone in the two other specimen. The longitudinal crest is complete. The protocone is either crest-shaped or cusp-like. The sinus is curved anteriorly. The 1st syncline is absent; the 2nd is small and closed in two specimens; the 3rd syncline is open labially and the 4th syncline is closed.

M1 – all specimens are large and the outline is square-shaped. The longitudinal crest is well-developed in one specimen, constricted and weak in two and interrupted in one specimen. The sinus is curved forward. The mesoloph is long and connects to the paracone in two specimens; in two others it is interrupted before the paracone. The metaloph runs parallel to the long posteroloph. The 1st syncline is always closed; the 2nd syncline is labially closed (2/4) or open (2/4); the 3rd syncline is open and the 4th syncline is closed.

M2 – the teeth are slightly smaller and narrower than M1. The longitudinal crest is complete; the mesoloph either connects to the paracone or ends labially free. The 1st and 4th synclines are closed and the sinus is anteriorly curved.

d4 – the teeth are elongated with an oval anterior outline and flat occlusal surface. The longitudinal crest is complete and connects to the middle part of the hypolophid. The hypoconid is crest-shaped and posteriorly oriented. The 1st and 2nd synclinids are closed, the 3rd synclinid is lingually open and the 4th synclinid is closed in one specimen, while lingually open in the second. The sinusid is directed posteriorly.

m1 – the teeth are elongated, but less so than d4. The outline of the teeth is sub-rectangular, narrower in its anterior part. The longitudinal crest is complete. The 1st, 2nd and 4th synclinids are closed while the 3rd is wide and lingually open. The 1st synclinid is the narrowest. The sinusid is directed posteriorly and the hypoconid is elongated.

m2 – one of available specimens has damage in its anterior part, missing the anterolophid. The outline is similar to the m1, but narrower in the posterior part. Both specimens have a complete longitudinal crest. The mesolophid is long and connects to the metaconid. In both specimens the mesolophid is constricted close to the longitudinal crest; thus, 2nd and 3rd synclinids are connected. The posterolophid is long and oblique. The 1st, 2nd and 4th synclinids are lingually closed.  

Remarks: see remarks in *Keramidomys* sp  

*Keramidomys* sp.  

(Fig. 2I-L)  

Material: 1 p4 (TB170102, 0.71 × 0.74), 1 m1/2 (TB107103, 0.76 × 0.81), 4 m3 (TB170110, 0.59 × 0.67; TB107111, 0.68 × 0.71; TB107112, 0.65 × 0.68; TB107113, 0.66 × 0.71).

Description: p4 – the outline of the tooth is oval and narrow in its anterior part. The tooth has small damage in the protocone area. The anterolophid is almost fused with the metalophid. The mesolophid is short, directed anteriorly and connects to the metalophid enclosing a very small 2nd syncline which is placed close to the longitudinal axis of the tooth. The longitudinal crest is complete. The 3rd synclinid and sinusid are equally developed. The 4th synclinid is closed.

m1/2 – the shape of outline is sub-rectangular. Four lophids are developed – anterolophid, metalophid, hypolophid and posterolophid. The longitudinal crest is transverse and connects protocone to the middle of the hypolophid. The 1st synclinid is very narrow, while the 4th synclinid is well developed. Both synclinids are closed.

m3 – three specimens have developed four main lophids, while one has only three. The mesolophid is missing and the longitudinal crest is complete. In two specimens, the anterolophid and the metalophid are close to each other forming closed and very narrow 1st synclinid. In one specimen (TB107112) these lophids are fused to one. Due to the missing mesolophid, only the large synclinid is developed between the metalophid and the hypolophid. The 4th syncline is lingually open in one specimen (TB107112), while it is closed in three others. One specimen (TB107113) has the anterolophid and metalophid fused similarly as in TB107112, but has developed a small rounded synclinid in the antero-lingual part of the occlusal surface formed by posteriorly directed spur from the metalophid, metaconid and by remnant of the mesolophid that is detached from the longitudinal spur. The sinusid is transverse or directed posteriorly.

Remarks: The material attributed to *Keramidomys ermannorum* fits well in size and morphology with the type material described by Daxner-Höck & Höck [9] from Richardhof – Golfplatz (MN9). Daxner-Höck & Höck [2,9] described main evolutionary trends in *K. ermannorum* from various Austrian localities ranging from MN9 to MN11. These trends are development of lophodonty, deeper sinus (id) in younger populations, flat occlusal surface, labial and lingual connections of lophs (ids) and wider molars. Considering these evolutionary trends, our teeth attributed to *K. ermannorum* show more advance features such as a labial and lingual connection of lophs (ids), lophodont character of flat occlusal surface, wide lower molars and generally larger size. Our population can be therefore morphologically compared with younger occurrences dated as MN11 (Kohfidisch and Eichkogel).

The few lower molars attributed to *Keramidomys* sp. show significant reduction of lophids. Similar reduced dental pattern was described from Richardhof – Golfplatz (MN9) by Daxner-Höck & Höck [2,9] and they attributed these teeth to *K.* cf. *pertesunatoi*. The premolar, m1/2 and all third lower molars from Triblavina show reduction of the lophids possessing only four or even three main lophids and the presence of a complete longitudinal crest in all specimens. The only described and figured m3 of *K. pertesunatoi* from its type locality Can Llobateres 1 [10] shows different dental pattern than our specimens but the figured m2 [10]; Fig. 5g] is very similar to our specimen TB170103. On the other hand, our lower premolar shows morphological similarities with Austrian material from Richardhof-Golfplatz. Thus, *Keramidomys* with a reduced dental pattern from Triblavina seems to be related to older Austrian population. Until larger collections will be available to confirm this hypothesis, the material from Triblavina is best classified as *Keramidomys* sp.

## 2 Experimental design, materials, and methods

The artificial temporal outcrop was accessible during construction of the road near Triblavina [1] in June 2017. At least 2.5 tons of sediment from the fossiliferous layer was dried, soaked and washed on a set of stable sieves [11]. The mesh size of the lower sieve was 0.5 mm and the residue was not treated with acids for sake of preserving mollusc shells. Fossil remains were then picked manually under the stereo-microscope. More than 170 teeth of small mammals were recovered. The rodent assemblage consists of 87 identifiable teeth and are described in detail here. Insectivores were also found and collected and are currently under study. Beside mammals, also molluscs were picked from the washed residuum and the composition of the assemblage is also discussed here.

The material described here is stored in the collections of Department of Geology and Paleontology, Comenius University in Bratislava, Slovakia. The upper cheek teeth are indicated by upper case (P4, M1, M2, M3) and lower cheek teeth by lower case (p4, m1, m2, m3). Where distinction between first and second molars is questionable, these are indicated as m1/2 or M1/2. Teeth measurements in mm were taken by calibrated micrometer eyepiece on a Leica MZ75 stereomicroscope. The provided cheek teeth measurement are the occlusal surface maximum length (L) and width (W), unless otherwise stated. Terminology used for teeth parts follows [12] for Murinae [13]; for Cricetidae [14]; for Gliridae [7]; for Eomyidae; and [15] for Sciuridae. Taxa were studied by direct comparison with specimens held at the Department of Geology and Paleontology, Comenius University in Bratislava and from published data of type materials.
